# Effects of Growth Hormone (GH) Deficiency and GH Replacement Therapy on Health-Related Physical Fitness in Children

**DOI:** 10.1210/clinem/dgaf676

**Published:** 2025-12-20

**Authors:** Nicola Improda, Giada Ballarin, Donatella Capalbo, Fabiana Santamaria, Giuliana Valerio, Luca Scalfi, Mariacarolina Salerno

**Affiliations:** Neuroendocrine Diseases and Obesity Unit, Department of Neuroscience, Santobono-Pausilipon Children's Hospital, Naples 80122, Italy; Pediatric Endocrinology Unit, Department of Translational Medical Sciences, Federico II University of Naples, Naples 80131, Italy; Neuroendocrine Diseases and Obesity Unit, Department of Neuroscience, Santobono-Pausilipon Children's Hospital, Naples 80122, Italy; Department of Medical, Movement and Wellbeing Sciences, Parthenope University of Naples, Naples 80133, Italy; Clinical Research Center DEMeTra, Department of Translational Medical Sciences, Federico II University of Naples, Naples 80131, Italy; Pediatric Endocrinology Unit, Department of Mother and Child, University Hospital Federico II, Naples 80131, Italy; Pediatric Endocrinology Unit, Department of Mother and Child, University Hospital Federico II, Naples 80131, Italy; Department of Medical, Movement and Wellbeing Sciences, Parthenope University of Naples, Naples 80133, Italy; Department of Public Health, Federico II University of Naples, Naples 80131, Italy; Pediatric Endocrinology Unit, Department of Translational Medical Sciences, Federico II University of Naples, Naples 80131, Italy; Pediatric Endocrinology Unit, Department of Mother and Child, University Hospital Federico II, Naples 80131, Italy

**Keywords:** growth hormone deficiency, muscle strength, body composition, health-related fitness, cardiorespiratory fitness

## Abstract

**Context:**

The effects of growth hormone deficiency (GHD) and GH replacement therapy (GHRT) on health-related physical fitness are still unclear.

**Objective:**

This work aimed to characterize the effects of GHD and GHRT on health-related physical fitness in children.

**Methods:**

We enrolled 49 GHD children (aged 10.7 ± 1.9 years) and 46 controls (aged 11.8 ± 2.3 years). Both groups underwent baseline assessment of body composition, muscle strength, flexibility, cardiorespiratory fitness, insulin-like growth factor-1, metabolic equivalents (METs-min/wk), sitting time, and health-related quality of life (HRQoL). These parameters were reevaluated in GHD patients after 1-year GHRT.

**Results:**

Compared to controls, GHD patients exhibited higher sitting time (3603.5 ± 1334.8 min/wk vs 3011.6 ± 1215.3 min/wk; *P* = .03), and waist-to-height ratio (WtHR) (0.50 ± 0.07 vs 0.45 ± 0.06; *P* = .001), reduced muscle strength (squat jump 13.88 ± 4.0 vs 15.7 ± 4.2 cm; *P* = .04; long jump 114.6 ± 25.8 vs 126.2 ± 29.6 cm; *P* = .04; counter-movement jump 13.7 ± 3.9 vs 16.4 ± 4.7 cm; *P* = .002), 6-minute walking distance (6MWD) (501.1 ± 69.3 vs 535.3 ± 65.6 m; *P* = .03), and maximal oxygen uptake (VO_2_max) (29.7 ± 4.7 vs 31.9 ± 4.6 mL/kg/min; *P* = .02). One-year GHRT led to reduced sitting time (3107.5 ± 1370.9 min/wk; *P* = .03), WtHR (0.45 ± 0.06; *P* = .02) and fat mass percentage (27.4 ± 5.4 vs 23.7 ± 5.8; *P* < .0001), increased METs-min/wk (2174.5 ± 1737.9 vs 2788.1 ± 2395.0; *P* = .05), fat-free mass (22.1 ± 7.6 vs 27.9 ± 9.4 kg; *P* < .0001), handgrip strength (11.1 ± 3.6 vs 15.4 ± 4.9 kg; *P* = .0001) squat jump (16.6 ± 4.7 cm; *P* = .01), long jump (129.6 ± 25.2 cm; *P* < .0001), countermovement jump (16.0 ± 4.3 cm; *P* = .003), 6MWD (540.5 ± 88.5 m; *P* = .01), VO_2_max (31.8 ± 5.1 mL/kg/min, *P* = .02), and physical (76.76 ± 16.42 vs 84.61 ± 11.63; *P* < .007) and psychosocial HRQoL scores (75.61 ± 15.12 vs 80.91 ± 11.12; *P <* .05).

**Conclusion:**

GHRT exerts broad beneficial effects on health-related physical fitness and HRQoL in children with GHD.

The primary function of growth hormone (GH) in children is to promote linear growth; however, it also has beneficial effects on numerous metabolic and nonmetabolic aspects (1-3). Indeed, GH deficiency (GHD) can be associated with several cardiovascular and metabolic alterations already in childhood (4-7).

The effects of GHD and GH replacement therapy (GHRT) on functional outcomes, such as physical fitness and functioning or psychosocial aspects, which are key determinants of patient well-being, remain largely unexplored (3).

Health-related physical fitness is a multicomponent concept including different dimensions (body composition, musculoskeletal fitness, cardiorespiratory fitness, and flexibility) that leads to the ability to perform daily activities with vigor, while maintaining specific capacities or skills associated with a low risk of developing chronic diseases and premature death (8).

A role for endogenous GH in physical fitness was first proposed decades ago (9-11). Observations showed that increased GH concentrations during exercise led to elevate the levels of free fatty acids, thereby enhancing the availability of oxidizable fat for exercising muscles and prolonging exercise capacity (12, 13). GH might also influence physical fitness through anabolic modifications of various organs and systems, including improved cardiac performance, body composition, increased muscle mass and strength, and more efficient thermoregulation (3). Additionally, GH has been shown to play indirect (14) and direct (15, 16) actions in regulating skeletal muscle protein synthesis, which might lead to increasing muscle mass. Specifically, short-term GH treatment stimulates protein and muscle synthesis, while its longer-term benefits seem to rely more heavily on insulin-like growth factor-1 (IGF-1)'s antiproteolytic effects (17).

In keeping with this, some studies in GHD adults have reported reduced isometric and isokinetic muscle strength (18-21), while others have found that jumping capacity, postural (quadriceps), and nonpostural (handgrip) strength were comparable to controls (22, 23). Although, according to a recent meta-analysis, muscle strength was not improved by short-term GHRT (24), a prospective study reported an increase in this parameter during long-term GHRT (25). Moreover, cardiorespiratory fitness is significantly impaired in adults with GHD, as demonstrated by a reduction of maximal oxygen uptake (VO_2_max) up to 27% compared to controls (26), which significantly benefits from short-term GHRT (17, 27, 28) These findings were also highlighted by 2 recent meta-analyses including 268 patients from 11 studies (29) and 306 patients from 15 studies (30), respectively.

Data in children regarding these parameters are scanty. We have recently documented that GHD adolescents may exhibit lower VO_2_max compared to healthy controls (5), which can be restored by 1-year GHRT, but our study lacked data regarding changes in other components of health-related physical fitness that may be influenced by GHD (5).

With regard to muscle strength, only one pediatric study has been published so far, showing that prepubertal boys with GHD may have weaker muscles and less endurance than healthy controls (31), which improve after 1-year GHRT. However, this study was limited by the very small size and included a heterogeneous cohort of either GHD or idiopathic short stature (ISS) individuals.

The aim of the present study was to characterize different domains of health-related physical fitness (musculoskeletal fitness, cardiorespiratory fitness and flexibility, and body composition) in children with GHD. In addition, we explored whether GHRT exerted beneficial effects on the outcomes previously described, as well as on lifestyle habits (eg, physical activity levels and sedentary time) and health-related quality of life (HRQoL) during a follow-up of 1 year.

## Materials and Methods

### Patients

Forty-nine children with untreated isolated GHD (31 boys, 63%) aged 10.7 ± 1.9 years (range, 7-14.5 years) have been enrolled in the study. Diagnosis of GHD was made according to clinical and auxological criteria associated with insufficient GH response (peak GH <8 µg/L) after 2 stimulation tests (32, 33). All the patients started GH at a dose of 25 µg/kg/day. Magnetic resonance imaging of the hypothalamus-pituitary region showed a normal gland in 35 patients and structural abnormalities in 14 patients, including pituitary hypoplasia (n = 12) and/or empty sella (n = 4) and/or pituitary cyst (n = 1).

Mean GH peak after arginine was 4.25 ± 2.05 µg/L, while it was 4.53 ± 1.8 after glucagon stimulation.

At study entry, only 3 out of 49 (6%) GHD patients had entered puberty (Tanner stage 2), and another 3 participants started puberty during the study.

Exclusion criteria were the presence of concomitant chronic diseases and/or genetic syndromes.

### Controls

Forty-six healthy individuals comparable for age (11.8 ± 1.9 years; range, 7.5-14.3 years), sex (25 boys, 54%), pubertal status, and body mass index (BMI) participated in the study as controls. These participants were referred to the Pediatric Endocrinology unit for short stature and were diagnosed, following appropriate assessment, as having familial short stature or constitutional delay of growth and puberty.

### Study Design

The study has 2 arms: first, a case-control cross-sectional study and second, a 1-year prospective study in the GHD group.

At study entry, both patients and controls underwent evaluation of anthropometric measures, systolic (SBP) and diastolic (DBP) blood pressure, body composition, musculoskeletal fitness, cardiorespiratory fitness, flexibility, and serum IGF-1 concentrations. Height, weight, and waist circumference were measured by the same investigator (G.B.). A mechanical column scale (Seca 711) and a Harpenden stadiometer were used to measure body weight (to the nearest 0.1 kg) and height (to the nearest 0.5 cm), respectively.

BMI was calculated by dividing weight in kilograms by height in meters squared. Height and BMI were standardized for age and sex and expressed as SD score (SDS) according to the Italian reference standards (34). Blood pressure was measured after at least 10 minutes’ resting with an aneroid sphygmomanometer and registered as the mean value of 3 measurements.

IGF-1 concentrations were expressed as SDS according to the normative data provided by the manufacturer.

Moreover, the Italian version of the International Physical Activity Questionnaire (IPAQ)-short form was self-administered with parental supervision, providing metabolic equivalents (METs-min/wk), estimating the metabolic cost (energy expenditure as reflected by oxygen consumption) of physical activity, as well as an average number of hours spent in a sitting position daily (sitting time) (35). It asks about the frequency (days per week), duration (minutes or hours per day), and intensity (vigorous, moderate, or walking) of physical activity performed in the last 7 days, and consists of various domains, including school-related physical activity, household activities, transportation, recreation, sport, and leisure time, and time spent sitting (35).

HRQoL was assessed using the Pediatric Quality of Life Inventory version 4.0 (PedsQL 4.0), a validated instrument encompassing 23 items grouped into 4 main domains (physical functioning, emotional functioning, social functioning, and school functioning) that provides separate scores (on a scale from 0 to 100, with higher scores indicating better HRQoL) for physical health and psychosocial health (sum of emotional functioning, social functioning, and school functioning) (36). It was administered to the child or his or her parents by the same trained psychologist in accordance with the standardized procedures provided by the developers (36).

All these parameters were reassessed only in GHD individuals after 1 year of GH treatment.

The study was approved by our institutional ethical committee (reference No. 227/20). Informed consent for participation in the study was obtained from all the patients and/or their families.

### Body Composition

Waist circumference was measured in the standing position with a nonelastic tape placed at the midpoint between the lower rib margin and the iliac crest. Hip circumference was measured at the level of the widest portion of the trochanters. Waist-to-hip ratio (WHR) and waist-to-height ratio (WHtR) were then computed and used to evaluate visceral adiposity. The maximal calf circumference was measured on the right calf by moving the measuring tape along the calf until the maximum circumference was identified (37). Arm and forearm circumferences were measured with the participant standing upright with the arms hanging loosely, by placing the measuring tape at the crossed point perpendicular to the long axis of the upper right arm and forearm, respectively (37). Biceps and triceps skinfold thickness were measured with a Harpenden plicometer and the mean between the 2 sides was used for comparison between groups. All these measurements were taken by the same well-trained operator and expressed in centimeters to the nearest 0.1 cm, except for skinfold thickness, which was expressed in millimeters to the nearest 0.2 mm.

Arm muscle area (AMA), total arm area (ATA), and arm fat area (AFA) were calculated according to the following formulas: AMA (cm^2^) = (arm circumference – π × triceps skinfold thickness)^2^/4π; ATA (cm^2^) = π/4 × (arm circumference/π)^2^ (π = 3:1416); AFA (cm^2^) = ATA − AMA (37).

Multifrequency bioimpedance analysis (BIA) was performed with a tetrapolar technique in standardized conditions (ambient temperature 23 °-25 °C, fasting >3 hours, empty bladder, supine position ≥10 minutes). Patients were asked to lie down with their legs and arms slightly abducted at 30°. Z and phase angle were measured at 50 kHz both for the dominant and nondominant side of the body injecting an electrical alternating current of 800 mA. Fat-free mass (FFM) and total body water (TBW) were predicted from BIA data using the available predictive equations for children. Fat mass (FM) was calculated as body weight minus FFM, and FM% was calculated as the ratio of FM to body weight (38).

### Muscle Strength

Muscle strength was assessed through an extensive battery of tests (4). Before each test, participants were permitted one practice trial to familiarize themselves with the procedure.

- Hand-grip strength was measured isometrically at the dominant side using a dynamometer with adjustable grip (TKK 5101 Grip D; Takey). Participants were asked to carry out 3 maximal efforts, lasting 4 to 5 seconds, with a 2-minute interval in between, and encouraged to improve their previous scores. Only the highest value was retained for analysis.
- Squat jump height (cm) was used to assess the explosive strength of lower limbs (39). The OptoJump device (MicroGate), which consists of photoelectric cells placed on 2 parallel bars (1 transmitter and 1 receiver unit, each measuring 100 × 4 × 3 cm, 1.5 kg weight) was positioned on the floor and connected to a personal computer. After familiarizing themselves with the device, the participants were instructed on how to perform the squat jump. Participants started from the standing position with their hands on their hips; they were then invited to flex their knees and hold a predetermined knee position (∼90°) until the acoustic signal started. At that point, they were invited to jump with maximal effort without performing any countermovement phase and without moving their hands. The distance in centimeters was measured from the take-off line to the point where the back of the heel nearest to the take-off line lands on the ground. The best score of 2 repeated tests was considered.
- Countermovement jump height (cm) was measured with the OptoJump device (MicroGate) (as described earlier) to assess the explosive force of the lower limbs (39). Participants were asked to perform a single jump starting from an upright position with hands on hips and with counter movement. The jump was performed as follows: rest hands on hips (to measure leg performance instead of arm performance); stand straight up for 1 to 2 seconds; jump as high as possible; land with normal flexion; and stand still in a neutral position for 1 to 2 seconds. The highest of 3 jumps was used for analysis.
- Total strength composite score was created by summing strength values of upper- (hand-grip strength expressed in kilograms) and lower-limb (jump capacity expressed in centimeters) strength, to practically capture both upper- and lower-body strength of each participant in a single value.
- Sit to stand test (STS)−30 seconds was used to assess leg strength and endurance. Participants were asked to sit with their feet shoulder width apart, flat on the floor, and their arms crossed at the wrists and held close to the chest. From the sitting position, they were invited to stand completely up, then completely back down, repeatedly for 30 seconds. The total number of complete chair stands was recorded (40).
- STS–5 repeats (STS-5R) was used to assess leg strength and balance. Participants were asked to repeat the sit-to-stand action 5 times as quickly as possible, and the time taken to complete the 5 repetitions was recorded (40).

### Body Flexibility and Functional Mobility

- Timed Up and Go was used to assess functional mobility and balance. Each participant was invited to stand up and walk at a comfortable speed to the end of the tape, turn around, walk back to the chair, and sit down. The final measure was the mean of 3 trials, separated by 2-minute rest intervals (41).
- Sit and reach was used to assess body flexibility. Participants were asked to sit on the floor with legs stretched out straight ahead, without shoes. The soles of the feet were placed flat against the sit and reach box, with both knees locked and pressed flat to the floor (if needed, assisted by the tester). With palms facing downward, and hands on top of each other or side by side, the participants were invited to reach forward along the measuring line as far as possible, with the hands at the same level (4).
- Gait speed: Participants were asked to walk along a 4-meter path at a comfortable pace, and the time (seconds) taken to complete the path was recorded and used to calculate gait speed as 4(m)/time(seconds) (42).

### Cardiorespiratory Fitness

This outcome was assessed through the 6-minute walking test (6MWT), about 1 hour after completing the battery of muscle functional tests. Participants were individually invited to walk on a 30-m, flat, straight corridor and the distance walked (6MWD) was measured (43). During the test, all participants were encouraged with standardized phrases (eg, “keep going,” “you are doing well”) and/or by informing them regarding the remaining time. VO2max was estimated using the following multiple regression equation: VO_2_max (mL/kg/min) = 12.701 + (0.06 × 6MWD in m) − (0.732 × body mass index) (44).

### Statistical Analysis

Statistical analysis was performed using the software SPSS (SPSS Statistics for Windows, version 26.0, IBM Corp). Quantitative variables were reported as mean and SD. Categorical variables were reported as number (n) and percentage (%). *T* tests were used to compare study end points between the 2 groups (GHD patients vs healthy controls) at baseline. Analysis of variance for repeated measures was used to assess the effect of GH treatment. Each study participant acted as their own control, allowing for within-group comparisons of the mean change from baseline at 12 months. Bivariate analysis was used to explore the relationship between 2 variables. Statistical significance was set at 5%.

## Results

General features of our study cohorts are summarized in Table 1. At study entry the average height SDS of the patients was slightly lower than controls (−2.1 ± 0.6 vs −1.8 ± 0.55; *P* < .001), while it improved significantly after 1 year of GHRT (*P* = .001) (see Table 1). Baseline BMI SDS, SBP, and DBP were comparable between patients and controls and did not significantly change after 1 year of GHRT (see Table 1).

**Table 1.** General characteristics and quality of life of growth hormone deficiency patients and controls at study entry and after 1 year

	Baseline	1 y	*P^a^*	*P^b^*
Sample size				
GHD	49	49	NA	NA
Controls	46	NA		
Age, y				
GHD	10.7 ± 1.9	11.8 ± 1.9	NS	NS
Controls	11.1 ± 2.3	NA		
Male n (%)				
GHD	31 (63%)	31 (63%)	NS	NS
Controls	25 (54%)	Na		
Height (SDS)				
GHD	−2.1 ± 0.6	−1.5 ± 0.7	<.001	<.001
Controls	−1.8 ± 0.55	NA		
BMI (SDS)				
GHD	−0.5 ± 1.3	−0.7 ± 1.1	NS	NS
Controls	−0.7 ± 1.1	NA		
SBP, mm Hg				
GHD	103.95 ± 11.13	105.07 ± 9.81	NS	NS
Controls	98.36 ± 8.51	NA		
DBP, mm Hg				
GHD	69.47 ± 7.62	71.64 ± 8.81	NS	NS
Controls	66.14 ± 7.11	NA		
IGF-1 (SDS)				
GHD	−1.2 ± 0.8	0.4 ± 0.8	<.0001	<.0001
Controls	−0.2 ± 0.9	NA		
METS, min/wk				
GHD	2174.5 ± 1737.9	2788.1 ± 2395.0	.08	<.05
Controls	2902.8 ± 2355.2	NA		
Sitting time, min/wk				
GHD	3603.5 ± 1334.8	3107.5 ± 1370.9	.03	.03
Controls	3011.6 ± 1215.3	NA		
HRQoL Physical score				
GHD	76.76 ± 16.42	84.61 ± 11.63	NS	<.007
Controls	81.16 ± 12.36	NA		
HRQoL Psychosocial score				
GHD	75.61 ± 15.12	80.91 ± 11.12	NS	<.05
Controls	78.48 ± 13.13	NA		

Data are expressed as mean ± SD.

Abbreviations: BMI, body mass index; DBP, diastolic blood pressure; GH, growth hormone; GHD, growth hormone deficiency; HRQoL, health-related quality of life; IGF-1, insulin-like growth factor-1; METs, metabolic equivalents; NA, not available; NS, not significant; SBP, systolic blood pressure.

^*a*^ *P*, baseline GHD vs baseline controls.

^*b*^ *P*, baseline GHD vs 1-year GH replacement.

Baseline IGF-1 SDS was significantly lower in patients compared to controls (−1.2 ± 0.8 vs −0.2 ± 0.9; *P* = .0001) and significantly increased after 1 year of GH treatment (0.4 ± 0.8; *P* = .0001) (see Table 1).

Physical activity level, as measured by METs-min/week, was lower in GHD patients compared to healthy controls even though the difference did not reach statistical significance (2174.5 ± 1737.9 vs 2902.8 ± 2355.2 min/wk; *P* = .08), while a significant increase was observed after 1 year of GHRT (2788.1 ± 2395.0; *P* = .045). Moreover, untreated GHD patients exhibited more sedentary behavior compared to healthy controls (sitting time 3603.5 ± 1334.8 vs 3011.6 ± 1215.3 min/wk; *P* = .03), which significantly improved after 1 year of treatment with GH (3107.5 ± 1370.9; *P* = .03) (see Table 1).

Data regarding HRQoL revealed both physical and psychosocial health total scores comparable between patients and controls. However, compared to baseline scores, a statistically significant improvement was observed in GHD individuals after 1 year of GHRT both in physical (76.76 ± 16.42 vs 84.61 ± 11.63; *P* < .007) and psychosocial health total scores (75.61 ± 15.12 vs 80.91 ± 11.12; *P* < .05) (see Table 1).

### Body Composition

WHR was comparable between patients and controls at baseline (0.94 ± 0.04 vs 0.92 ± 0.06), and it significantly decreased (*P* = .02) after 1 year of replacement therapy (Table 2). WtHR was higher at baseline in GHD patients compared to controls (0.50 ± 0.07 vs 0.45 ± 0.06; *P* = .001), but it significantly decreased (0.45 ± 0.06; *P* = .02) after treatment, becoming similar to the control group (see Table 2).

**Table 2.** Body composition of growth hormone deficiency patients and controls at study entry and after 1 year

	Baseline	1 y	*P^a^*	*P^b^*
WHR				
GHD	0.94 ± 0.04	0.86 ± 0.05	NS	.02
Controls	0.92 ± 0.06			
WtHR				
GHD	0.50 ± 0.07	0.45 ± 0.06	.001	.02
Controls	0.45 ± 0.04	NA		
FFM, kg				
GHD	22.1 ± 7.6	27.9 ± 9.4	NS	<.0001
Controls	24.6 ± 8.6	NA		
FM, %				
GHD	27.4 ± 5.4	23.7 ± 5.8	NS	<.0001
Controls	25.9 ± 5.4	NA		
TBW, L				
GHD	13.9 ± 4.0	18.1 ± 5.6	NS	<.0001
Controls	15.1 ± 4.4	NA		
Bicipital folds, mm				
GHD	8.5 ± 4.4	7.7 ± 4.5	NS	NS
Controls	8.6 ± 4.8	NA		
Tricipital folds, mm				
GHD	12.2 ± 5.6	11.0 ± 5.3	NS	NS
Controls	12.5 ± 5.1	NA		
AMA, mm^2^				
GHD	32.2 ± 13.4	35.1 ± 13.4	NS	.03
Controls	33.1 ± 11.2	NA		
AFA, mm^2^				
GHD	1.3 ± 0.8	1.2 ± 0.8	NS	NS
Controls	1.3 ± 0.7	NA		
ATA, mm^2^				
GHD	33.9 ± 14.2	36.0 ± 14.1	NS	NS
Controls	34.0 ± 11.6	NA		

Data are expressed as mean ± SD.

Abbreviations: AFA, arm fat area; AMA, arm muscle area; ATA, arm total area; FFM, fat-free mass; FM, fat mass; GH, growth hormone; GHD, growth hormone deficiency; NA, not available; NS, not significant; TBW, total body water; WHR, waist-to-hip ratio; WtHR, waist-to-height ratio.

^*a*^ *P*, baseline GHD vs baseline controls.

^*b*^ *P*, baseline GHD vs 1-year GH replacement.

**Figure 1.**
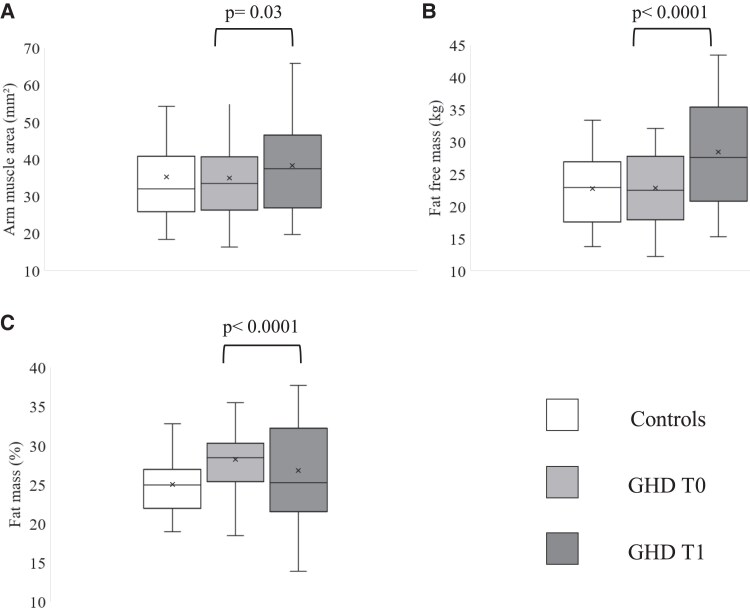
Body composition parameters: A, arm muscle area; B, fat-free mass; and C, fat mass percentage before and after 1-year growth hormone (GH) replacement therapy in GH deficiency (GHD) patients, compared with controls.

At study entry, patients with GHD exhibited higher values of FM% (27.4 ± 5.4 vs 25.9 ± 5.4%), and lower values of FFM (22.1 ± 7.6 vs 24.6 ± 8.6 kg), TBW (13.9 ± 4.0 vs 15.1 ± 4.4 L), and AMA (32.2 ± 13.4 vs 33.1 ± 11.2 mm^2^), compared to controls, though not statistically different **(**Fig. 1A-1C**)**. After 1 year of GHRT, FFM, TBW, and AMA significantly increased (27.9 ± 9.4 kg; *P* < 0.0001; 18.1 ± 5.6 L; *P* < .0001; and 35.1 ± 13.4 mm^2^; *P* = .03, respectively) (see Fig. 1A and 1B) and FM% significantly decreased (23.7 ± 5.8%; *P* < .0001) in the patient group (see Table 2 and Fig. 1C).

Biceps and triceps skinfolds, ATA, and AFA were comparable to controls in GHD patients at study entry and after 1 year of GHRT (see Table 2).

### Muscle Strength, Body Flexibility, and Functional Mobility and Cardiorespiratory Fitness

**Figure 2.**
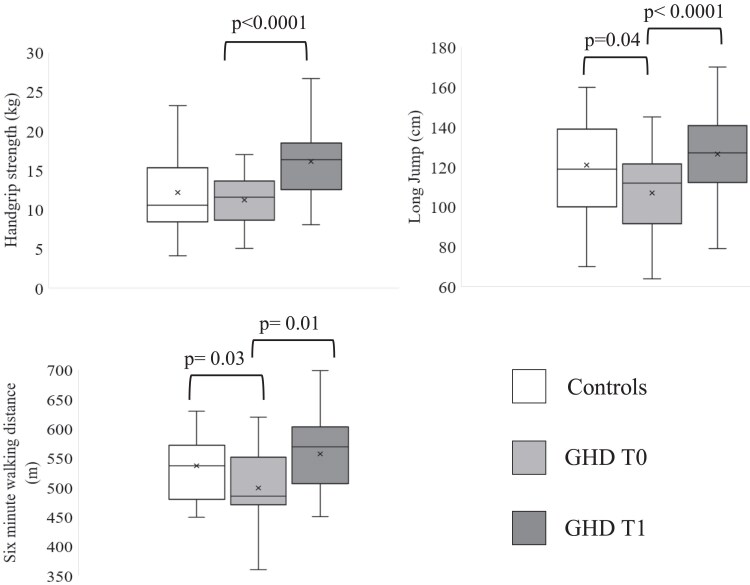
Muscle strength and cardiorespiratory fitness: A, handgrip strength; B, long jump; and C, 6-minute walking distance before and after 1-year growth hormone (GH) replacement therapy in GH deficiency (GHD) patients, compared with controls.

At baseline, handgrip strength (Fig. 2A), total strength, sit-to-stand 30 seconds, sit-to-stand 5 repeats, timed up and go, sit and reach, and gait velocity were similar between the 2 study groups, while jumping measures (long jump; *P* < .0001; squat jump; *P* = .01; and countermovement jump; *P* = .002) (Fig. 2B), 6MWD (*P* = .04) (Fig. 2C), and VO_2_max (0.02) were lower in GHD patients compared to controls (Table 3).

**Table 3.** Results of tests evaluating muscle strength, body flexibility and functional mobility and cardiorespiratory fitness of growth hormone deficiency patients and controls at study entry and after 1 year

	Baseline	1 y	*P^a^*	*P^b^*
Handgrip strength, kg				
GHD	11.1 ± 3.6	15.4 ± 4.9	NS	<.0001
Controls	11.9 ± 5.6	NA		
Squat jump, cm				
GHD	13.88 ± 4.0	16.6 ± 4.7	.04	.01
Controls	15.7 ± 4.2	NA		
Countermovement jump, cm				
GHD	13.7 ± 3.9	16.0 ± 4.3	.002	.003
Controls	16.4 ± 4.7	NA		
Long jump, cm				
GHD	114.6 ± 25.8	129.6 ± 25.2	.04	<.0001
Controls	126.2 ± 29.6	NA		
Total strength				
GHD	109.8 ± 4.1	113.9 ± 5.6	NS	<.0001
Controls	110.6 ± 6.1	NA		
Sit-to-stand 30 s				
GHD	20.3 ± 4.3	20.4 ± 4.0	NS	NS
Controls	18.6 ± 4.3	NA		
Sit-to-stand 5 repeats				
GHD	8.23 ± 2.23	7.32 ± 2.15	NS	.04
Controls	8.99 ± 2.18	NA		
Timed Up and Go, s				
GHD	6.4 ± 1.0	5.9 ± 0.8	NS	.002
Controls	6.2 ± 1.1	NA		
Sit and reach test, cm				
GHD	8.3 ± 7.7	10.7 ± 9.8	NS	NS
Controls	11.7 ± 9.4	NA		
Gait speed, m/sec				
GHD	1.3 ± 0.3	1.4 ± 0.2	NS	.001
Controls	1.3 ± 0.2	NA		
6MWD, m				
GHD	501.1 ± 69.3	540.5 ± 88.5	.03	.01
Controls	535.3 ± 65.6	NA		
VO_2_max, mL/kg/min				
GHD	29.7 ± 4.7	31.8 ± 5.1	.02	.02
Controls	31.9 ± 4.6			

Data are expressed as mean ± SD.

Abbreviations: 6MWD, 6-minute walking distance; GH, growth hormone; GHD, growth hormone deficiency; NS, not significant; NA, not available; VO_2_max, maximal oxygen uptake.

^*a*^ *P*, baseline GHD vs baseline controls.

^*b*^ *P*, baseline GHD vs 1-year GH replacement.

One-year GHRT was associated with an improvement in muscle strength (Fig. 2A and 2B) (handgrip; *P* = .0001; total strength; *P* < .0001; squat jump; *P* = .01; countermovement jump, *P* = .003; long jump; *P* = .03; sit-to-stand test-5 repeats; *P* = .04) and gait velocity (*P* = .0014) (see Table 3). Moreover, a statistically significant improvement both in 6MWD (*P* = .005) (see Fig. 2C) and VO_2_max (*P* = .02) was observed after 1 year of GH (see Table 3). Notably, in a multiple regression model, 1-year variations in age, sex, or Tanner stage did not significantly predict changes in the measured physical fitness parameters (handgrip strength, squat jump, countermovement jump, long jump, and 6MWD).

### Correlations

Changes in height SDS correlated positively with changes in METs-min/week (*r* = 0.42; *P* = .003), and negatively with sitting time/week (*r* = −0.32; *P* = .02).

Sitting time was negatively related to IGF-1 (*r* = −0.41; *P* = .005), and METs-min/week (*r* = −0.31; *P* = .035).

Changes in FFM correlated positively with changes in IGF1 (*r* = 0.38; *P* = .011), AMA (*r* = 0.37; *P* = .011), handgrip strength (*r* = 0.49; *P* < .001), total strength (*r* = 0.42, *P* = .004), 6MWD (*r* = 0.29; *P* = .05), VO_2_max (*r* = 0.29, *P* = .048), and negatively with sitting time/week (*r* = −0.27; *P* = .05).

Likewise, changes in AMA correlated positively with changes in long jump (*r* = 0.28; *P* = .05), 6MWD (*r* = 0.38; *P* = .008), and VO_2_max (*r* = 0.33; *P* = .023).

Changes in FM% correlated negatively with changes in IGF-1 concentrations (*r* = −0.39; *P* = .008).

Changes in squat jump maximum, long jump, 6MWD, and VO_2_max were significantly correlated with IGF-1 concentrations (*r* = 0.35; *P* = .047; *r* = 0.27; *P* = .05; *r* = 0.30; *P* = .04; and *r* = 0.33; *P* = .025, respectively).

## Discussion

This study comprehensively examined the effects of GHD and GHRT on health-related physical fitness, lifestyle habits, and QoL in pediatric patients, thereby addressing a critical gap in our understanding of how GHD and GHRT affect functional parameters most relevant to pediatric overall well-being.

Our results documented that GHD in childhood can be associated with impaired health-related physical fitness and reduced physical activity, which can be restored by GHRT, eventually leading to a better HRQoL.

The effects of GHD and GHRT on muscle strength are still largely unknown. A few studies in GHD adults have yielded conflicting results. While some studies reported reduced isometric and isokinetic muscle strength (18-21), others did not find statistically significant differences compared to controls (22, 23). However, patients who discontinue GHRT during the transition period may experience a reduction in isometric muscle contraction and muscle mass, or may fail to achieve the expected gains in muscle strength when compared to GH-sufficient healthy individuals (45). Furthermore, although a recent meta-analysis did not find a significant effect of GHRT on muscle strength over a short-term period of 6 months (24), a long-term study reported a progressive increase in most measures of isometric and isokinetic muscle strength over up to 7 years of GHRT (25). This suggests that improvements in muscle strength associated with GHRT may require long-term duration of treatment (25).

Data on muscle strength in children with GHD are lacking. GHRT in children born small for gestational age and with Prader-Willi syndrome positively affects muscle mass and physical fitness (46-49). A recent study examined the effect of GH in a cohort of prepubertal boys with ISS (n = 15) or GHD (n = 15), revealing a reduction in muscle strength and endurance compared to healthy controls (31) that improved after 12 months of GHRT. However, this study has several limitations, including a small sample size and the use of a threshold value for diagnosis of GHD at the higher end of the recommended range (peak GH responses to stimuli <10 ng/mL) (31). In this context, the results of our study provide clear evidence that untreated GHD may be associated with reduced muscle strength. Indeed, untreated GHD children showed worse performance in explosive strength tests (jumps), 6MWD, and VO_2_max. GHRT improved these measures along with handgrip, total strength, and functional mobility tests.

A crucial aspect that emerged from the study is the link between GH and body composition. GHD patients exhibited higher visceral adiposity evaluated through the WtHR, which significantly decreased during GHRT. Moreover, in line with the results of other pediatric studies (50-52), 1-year GHRT led to a significant increase in FFM, and AMA, with a decrease in FM%.

Given the significant increase observed in TBW, a well-known effect of GHRT (53, 54), parallel increases in AMA indicate that the changes in FFM are due to a genuine improvement in muscle mass, while fluid retention makes a smaller contribution (45). To our knowledge, AMA has never been directly compared between pediatric GHD patients and healthy controls. Two previous studies in adults evaluating this surrogate index of muscle mass at baseline (53, 54) have yielded conflicting results, with one (53) reporting significantly lower values of AMA, while the other’s (54) values were comparable to their respective controls. Consistent with our results, this latter study documented a significant increase in AMA during GHRT (54). The lack of redistribution of body fat in bicipital and tricipital skinfold thicknesses can be explained by previous findings in adults suggesting that GHD is associated with changes in subcutaneous fat prominently in the truncal regions (50).

Furthermore, our findings indicate that increased muscle mass is a key mediator for the improvement in muscle strength observed in patients receiving GHRT. This could be due to complex mechanisms mainly mediated by IGF-1, including increased protein synthesis, antiproteolytic effects, myocyte differentiation and muscle cell proliferation, and/or structural changes within myocytes (3, 17).

Handgrip strength and jumping represent valuable tools for the evaluation of upper-and lower-limb strength, and studies have shown a strong correlation between them, particularly in athletes (55). The discrepancy between cases and controls in the baseline measures of muscle strength, consisting of reduced baseline values only for jumping tests and no difference in the handgrip test, may have several explanations. First, forearm and hand muscles might be more resistant to the effects of GHD because they are less dependent on body mass compared to other muscle groups (21). Second, the reduced physical activity (56, 57) and/or the neuromuscular dysfunction (58) associated with GHD may have a more substantial effect on the complex, coordinated action of multiple muscle groups required for jumping, rather than on the isolated action required to squeeze a dynamometer. Third, GH has been shown to influence the anaerobic energy system, which is crucial for high-power, explosive movements like jumping (59, 60). Finally, it has been suggested that GH may promote the development of more type II muscle fibers (“fast-twitch”), which are responsible for power and speed (61) and thus its deficiency could lead to suboptimal muscle fiber composition for explosive power.

Data regarding VO_2_max derived from the 6MWT are consistent with our previous study, reporting that GHD children may exhibit lower VO_2_max compared to healthy controls, with improvement observed on starting GHRT (12). Although the cardiopulmonary exercise test remains the gold standard for directly and accurately measuring VO_2_max, the 6MWT is a submaximal test that provides a valuable assessment of functional capacity and exercise tolerance (62, 63).

Based on the 6MWD results, GHD children also seemed to have lower functional capacity and exercise tolerance than their peers, which are strong predictors of cardiovascular diseases (64, 65). GHRT is effective in ameliorating these parameters, which might, in turn, help to maintain the positive effects in body composition (11, 28, 66, 67).

The findings related to lifestyle behavior appear closely linked to the effects on health-related fitness. Patients with GHD showed lower levels of physical activity and a more sedentary lifestyle compared to controls. Notably, we could speculate that GHRT improves sedentary behaviors and METs-min/week through its effects on body composition and muscle mass. However, it is essential to consider that these behavioral improvements may not stem solely from direct hormonal effects on body composition and muscle mass, but may also reflect changes of a psychosocial or motivational nature intrinsically associated with the initiation of treatment (68).

So far, our study has been the first to evaluate sitting behavior, which is considered as an independent health risk factor for cardiovascular and metabolic diseases (69, 70). Furthermore, our data confirm the results of a previous pediatric study indicating positive effects of GHRT on METs min/week (71). It is worth noting that in the study by Alkan et al (71), METs were calculated directly from an exercise stress test on a treadmill, reflecting the individual's maximal cardiorespiratory functional capacity, rather than energy expenditure as METs-min/week estimated with the IPAQ questionnaire; however, a moderate correlation between IPAQ and objective measures of physical activity has been documented (72, 73).

Despite impairments in overall health-related fitness, our patients did not exhibit lower scores in psychosocial or physical HRQoL domains at baseline, in comparison to controls. However, it is worth noting that our control group included individuals investigated for short stature and/or growth deceleration and this may have affected their HRQoL scores (68). Data on this topic in untreated GHD children are scanty and inconclusive. While some studies reported lower baseline scores for patient-reported QoL in children with GHD compared to those with ISS (74), other findings indicate that QoL in untreated GHD children may be normal (73) or impaired only in specific subscales (75).

This disparity in results might be explained by the use of different tools, with only a minority of studies using questionnaires specifically developed for short-statured patients (73). However, it is worth noting that, during GHRT, our patients showed a significant increase in HRQoL scores, aligning with prior studies (76-79). While this demonstrates an important effect of GHRT on overall QoL, this HRQoL enhancement should be interpreted cautiously, as it may reflect psychosocial benefits from height gain or improved perceived health status, rather than being solely a direct biological effect of the hormone (73-76).

We acknowledge that our study has some limitations, including the relatively small sample size, the lack of information regarding dietary habits and pattern, energy and protein intake, engagement in sports, psychosocial and sociodemographic factors, and adherence to treatment. Crucially, the lack of 1-year follow-up data from the control group limits our ability to compare the magnitude of improvements in the GHD group due to GHRT with the expected natural changes in growth and pubertal development that would have occurred in the control cohort (eg, increased lean mass and strength). Similarly, the absence of comprehensive physical activity monitoring over the 1-year intervention period further prevents us from concluding that the observed improvements in muscle strength, flexibility, functional mobility, and cardiorespiratory fitness are attributable solely to GHRT, as these functional gains may have been partially influenced by an increase in the children's spontaneous exercise load.

A further methodological consideration pertains to body composition assessment. Indeed, despite BIA being a noninvasive, easy to use, and cost-effective technique used in several settings for the estimation of body composition compartments, its reliance for FFM and FM% estimations presents a potential limitation, as BIA estimates are susceptible to fluctuations in TBW (80). This is a relevant confounding factor during GHRT due to the known fluid-retention effects of GH (53, 54). Consequently, BIA might not be as sensitive as gold-standard methods like dual-energy x-ray absorptiometry (80). Conversely, AMA provides a useful surrogate index of muscle mass, as its calculation, derived from anthropometric measures (triceps skinfold and arm circumference), is less immediately affected by these acute hydration shifts. Hence, this relative stability of AMA helps mitigate the potential bias introduced by BIA in estimating changes in muscle mass in our patients undergoing GHRT.

### Conclusions

Our results demonstrate that GHD children exhibit impaired health-related physical fitness and reduced physical activity compared to their peers. GHRT provides considerable physical health benefits, including improved body composition and physical activity, enhanced muscle strength, cardiorespiratory fitness, and functional mobility. Although these results need to be confirmed in larger studies, they reinforce the concept that GHRT in childhood not only promotes linear growth, but also exerts beneficial effects on several physical and metabolic parameters. These improvements may, in turn, contribute to ameliorate long-term metabolic and cardiovascular outcomes and HRQoL in individuals affected by GHD.

## Data Availability

All datasets on which the conclusions of the paper rely are available to editors and reviewers on request. Data are not publicly available due to ethical reasons. Further enquiries can be directed to the corresponding author.

## Abbreviations

6MWD: 6-minute walking distance
6MWT: 6-minute walking test
AFA: arm fat area
AMA: arm muscle area
ATA: arm total area
BIA: bioimpedance analysis
BMI: body mass index
DBP: diastolic blood pressure
FFM: fat-free mass
FM: fat mass
GH: growth hormone
GHD: growth hormone deficiency
GHRT: growth hormone replacement therapy
HRQoL: health-related quality of life
IGF-1: insulin-like growth factor-1
IPAQ: International Physical Activity Questionnaire
ISS: idiopathic short stature
METs: metabolic equivalents
SBP: systolic blood pressure
STS: Sit to stand test
TBW: total body water
VO_2_max: maximal oxygen uptake
WHR: waist-to-hip ratio
WtHR: waist-to-height ratio

## References

[1] van der Sluis IM, Boot AM, Hop WC, De Rijke YB, Krenning EP, de Muinck Keizer-Schrama SM. Long-term effects of growth hormone therapy on bone mineral density, body composition, and serum lipid levels in growth hormone deficient children: a 6-year follow-up study. Horm Res. 2002;58(5):207‐214. 12401939 10.1159/000066262

[2] Moller N, Jorgensen JOL. Effects of growth hormone on glucose, lipid, and protein metabolism in human subjects. Endocr Rev. 2009;30(2):152‐177. 19240267 10.1210/er.2008-0027

[3] Improda N, Capalbo D, Esposito A, Salerno M. Muscle and skeletal health in children and adolescents with GH deficiency. Best Pract Res Clin Endocrinol Metab. 2016;30(6):771‐783. 27974190 10.1016/j.beem.2016.11.012

[4] Capalbo D, Barbieri F, Improda N, et al Growth hormone improves cardiopulmonary capacity and body composition in children with growth hormone deficiency. J Clin Endocrinol Metab. 2017;102(11):4080‐4088. 28938456 10.1210/jc.2017-00871

[5] Improda N, Moracas C, Mattace Raso G, et al Vascular function and intima-media thickness in children and adolescents with growth hormone deficiency: results from a prospective case-control study. Horm Res Paediatr. 2024;97(2):140‐147. 37290420 10.1159/000531473

[6] Capalbo D, MattaceRaso G, Esposito A, et al Cluster of cardiometabolic risk factors in children with GH deficiency: a prospective, case-control study. Clin Endocrinol. 2014;80(6):856‐862. 10.1111/cen.12393 24372071

[7] Esposito A, Capalbo D, De Martino L, et al Long-term effects of growth hormone (GH) replacement therapy on hematopoiesis in a large cohort of children with GH deficiency. Endocrine. 2016;53(1):192‐198. 26511947 10.1007/s12020-015-0781-9

[8] Ruiz JR, Castro-Piñero J, España-Romero V, et al Field-based fitness assessment in young people: the ALPHA health-related fitness test battery for children and adolescents. Br J Sports Med. 2011;45(6):518‐524. 20961915 10.1136/bjsm.2010.075341

[9] Hunter WM, Fonseka CC, Passmore R. The role of growth hormone in the mobilization of fuel for muscular exercise. Q J Exp Physiol Cogn Med Sci. 1965;50(4):406‐416. 5177005 10.1113/expphysiol.1965.sp001806

[10] Jenkins PJ . Growth hormone and exercise. Clin Endocrinol. 1999;50(6):683‐689. 10.1046/j.1365-2265.1999.00784.x 10468938

[11] Gibney J, Healy ML, Sonksen PH. The growth hormone/insulin like growth factor-I axis in exercise and sport. Endocr Rev. 2007;28(6):603‐624. 17785429 10.1210/er.2006-0052

[12] Widdowson WM, Healy ML, Sönksen PH, Gibney J. The physiology of growth hormone and sport. Growth Horm IGF Res. 2009;19(4):300‐307. 19505835 10.1016/j.ghir.2009.04.023

[13] Grandys M, Majerczak J, Kuczek P, Sztefko K, Duda K, Zoladz JA. Endurance training-induced changes in the GH-IGF-I axis influence maximal muscle strength in previously untrained men. Growth Horm IGF Res. 2017;32:41‐48. 28017505 10.1016/j.ghir.2016.12.003

[14] Green H, Morikawa M, Nixon T. A dual effector theory of growth-hormone action. Differentiation. 1985;29(3):195‐198. 3908201 10.1111/j.1432-0436.1985.tb00316.x

[15] Fryburg DA, Gelfand RA, Barrett EJ. Growth hormone acutely stimulates forearm muscle protein synthesis in normal humans. Am J Phys. 1991;260(3):E499‐E504. 10.1152/ajpendo.1991.260.3.E499 2003602

[16] Fryburg DA, Barrett EJ. Growth hormone acutely stimulates skeletal muscle but not whole-body protein synthesis in humans. Metabolism. 1993;42(9):1223‐1227. 8412780 10.1016/0026-0495(93)90285-v

[17] Woodhouse LJ, Mukherjee A, Shalet SM, Ezzat S. The influence of growth hormone status on physical impairments, functional limitations, and health-related quality of life in adults. Endocr Rev. 2006;27(3):287‐317. 16543384 10.1210/er.2004-0022

[18] Cuneo RC, Salomon F, Wiles CM, Hesp R, Sonksen PH. Growth hormone treatment in growth hormone-deficient adults. I. Effects on muscle mass and strength. J Appl Physiol. 1991;70(2):688‐694. 2022560 10.1152/jappl.1991.70.2.688

[19] Rutherford OM, Beshyah SA, Schott J, Watkins Y, Johnston DG. Contractile properties of the quadriceps muscle in growth hormone-deficient hypopituitary adults. Clin Sci 1995;88(1):67‐71. 10.1042/cs0880067 7705003

[20] Johannsson G, Grimby G, Sunnerhagen KS, Bengtsson BA. Two years of growth hormone (GH) treatment increase isometric and isokinetic muscle strength in GH-deficient adults. J Clin Endocrinol Metab. 1997;82(9):2877‐2884. 9284713 10.1210/jcem.82.9.4204

[21] Janssen YJ, Doornbos J, Roelfsema F. Changes in muscle volume, strength, and bioenergetics during recombinant human growth hormone (GH) therapy in adults with GH deficiency. J Clin Endocrinol Metab. 1999;84(1):279‐284. 9920096 10.1210/jcem.84.1.5411

[22] Sartorio A, Agosti F, De Col A, et al Muscle strength and power, maximum oxygen consumption, and body composition in middle-aged short-stature adults with childhood-onset growth hormone deficiency. Arch Med Res. 2008;39(1):78‐83. 18067999 10.1016/j.arcmed.2007.06.011

[23] Sartorio A, Narici M, Conti A, Monzani M, Faglia G. Quadriceps and hand-grip strength in adults with childhood-onset growth hormone deficiency. Eur J Endocrinol. 1995;132(1):37‐41. 7850008 10.1530/eje.0.1320037

[24] Widdowson WM, Gibney J. The effect of growth hormone (GH) replacement on muscle strength in patients with GH-deficiency: a meta-analysis. Clin Endocrinol. 2010;72(6):787‐792. 10.1111/j.1365-2265.2009.03716.x 19769614

[25] Götherström G, Elbornsson M, Stibrant-Sunnerhagen K, Bengtsson BA, Johannsson G, Svensson J. Ten years of growth hormone (GH) replacement normalizes muscle strength in GH-deficient adults. J Clin Endocrinol Metab. 2009;94(3):809‐816. 19088164 10.1210/jc.2008-1538

[26] Hartman ML, Weltman A, Zagar A, Qualy RL, Hoffman AR, Merriam GR. Growth hormone replacement therapy in adults with growth hormone deficiency improves maximal oxygen consumption independently of dosing regimen or physical activity. J Clin Endocrinol Metab. 2008;93(1):125‐130. 17956953 10.1210/jc.2007-1430

[27] Jørgensen JOL, Vahl N, Hansen TB, Thuesen L, Hagen C, Christiansen JS. Growth hormone versus placebo treatment for one year in growth hormone deficient adults: increase in exercise capacity and normalisation of body composition. Clin Endocrinol. 1996;45(6):681‐688. 10.1046/j.1365-2265.1996.8720883.x 9039333

[28] Cuneo RC, Salomon F, Wiles CM, Hesp R, Sönksen PH. Growth hormone treatment in growth hormone-deficient adults. II. Effects on exercise performance. J Appl Physiol. 1991;70:695‐700. 2022561 10.1152/jappl.1991.70.2.695

[29] Widdowson WM, Gibney J. The effect of growth hormone replacement on exercise capacity in patients with GH-deficiency: a meta-analysis. J Clin Endocrinol Metab. 2008;93(11):4413‐4417. 18697875 10.1210/jc.2008-1239

[30] Rubeck KZ, Bertelsen S, Vestergaard P, Jørgensen JOL. Impact of GH substitution on exercise capacity and muscle strength in GH-deficient adults: a meta-analysis of blinded, placebo-controlled trials. Clin Endocrinol. 2009;71(6):860‐866. 10.1111/j.1365-2265.2009.03592.x 19508603

[31] Malpani A, Welch L, Plummer D, et al Impact of growth hormone on skeletal muscle strength, power, endurance, and agility in prepubertal boys with short stature. J Clin Endocrinol Metab. 2025;110(12):3517‐3524. 40171942 10.1210/clinem/dgaf203

[32] Grimberg A, DiVall SA, Polychronakos C, et al Guidelines for growth hormone and insulin-like growth factor-I treatment in children and adolescents: growth hormone deficiency, idiopathic short stature, and primary insulin-like growth factor-I deficiency. Horm Res Paediatr. 2016;86(6):361‐397. 27884013 10.1159/000452150

[33] Hage C, Gan HW, Ibba A, et al Advances in differential diagnosis and management of growth hormone deficiency in children. Nat Rev Endocrinol. 2021;17(10):608‐624. 34417587 10.1038/s41574-021-00539-5

[34] Cacciari E, Milani S, Balsamo A, et al Italian cross-sectional growth charts for height, weight and BMI (6-20 y). Eur J Clin Nutr. 2000;56(2):171‐180. 10.1038/sj.ejcn.1601314 11857051

[35] Hagströmer, M., Bergman, P., De Bourdeaudhuij, I, et al Concurrent validity of a modified version of the International Physical Activity Questionnaire (IPAQ-A) in European adolescents: the HELENA study. Int J Obes. 2008;32(Suppl 5):S42‐S48. 10.1038/ijo.2008.182 19011653

[36] Varni JW, Seid M, Kurtin PS. The PedsQL™ 4.0: reliability and validity of the Pediatric Quality of Life Inventory™ version 4.0 generic core scales in healthy and patient populations. Med Care. 2001;39(8):800‐812. 11468499 10.1097/00005650-200108000-00006

[37] Abreo AP, Bailey SR, Abreo K. Associations between calf, thigh, and arm circumference and cardiovascular and all-cause mortality in NHANES 1999-2004. Nutr Metab Cardiovasc Dis. 2021;31(5):1410‐1415. 33762151 10.1016/j.numecd.2021.01.011

[38] Deurenberg P, van der Kooy K, Leenen R, Weststrate JA, Seidell JC. Sex and age specific prediction formulas for estimating body composition from bioelectrical impedance: a cross-validation study. Int J Obes. 1991;15(1):17‐25. 2010255

[39] Markovic G, Dizdar D, Jukic I, Cardinale M. Reliability and factorial validity of squat and countermovement jump tests. J Strength Cond Res. 2004;18(3):551‐555. 15320660 10.1519/1533-4287(2004)18<551:RAFVOS>2.0.CO;2

[40] Roldán-Jiménez C, Bennett P, Cuesta-Vargas AI. Muscular activity and fatigue in lower-limb and trunk muscles during different sit-to-stand tests. PLoS One. 2015;10(10):e0141675. 26506612 10.1371/journal.pone.0141675 PMC4624782

[41] Williams EN, Carroll SG, Reddihough DS, Phillips BA, Galea MP. Investigation of the timed ‘up & go’ test in children. Dev Med Child Neurol. 2005;47(8):518‐524. 16108451 10.1017/s0012162205001027

[42] Reychler G, Audag N, Dewulf S, Morale Mestre N, Caty G. Validation of 6 min step test and 4-m gait speed in children: a randomized cross-over study. Gait Posture. 2018;61:19‐24. 29289866 10.1016/j.gaitpost.2017.12.011

[43] ATS Committee on Proficiency Standards for Clinical Pulmonary Function Laboratories . ATS statement: guidelines for the six-minute walk test. Am J Respir Crit Care Med. 2002;166(1):111‐117. 12091180 10.1164/ajrccm.166.1.at1102

[44] Jalili M, Nazem F, Sazvar A, Ranjbar K. Prediction of maximal oxygen uptake by six-minute walk test and body mass Index in healthy boys. J Pediatr. 2018;200:155‐159. 29773305 10.1016/j.jpeds.2018.04.026

[45] Rutherford OM, Jones DA, Round JM, Buchanan CR, Preece MA. Changes in skeletal muscle and body composition after discontinuation of growth hormone treatment in growth hormone deficient young adults. Clin Endocrinol. 1991;34(6):469‐475. 10.1111/j.1365-2265.1991.tb00327.x 1889131

[46] Schweizer R, Martin DD, Schönau E, Ranke MB. Muscle function improves during growth hormone therapy in short children born small for gestational age: results of a peripheral quantitative computed tomography study on body composition. J Clin Endocrinol Metab. 2008;93(8):2978‐2983. 18505766 10.1210/jc.2007-2600

[47] Schweizer R, Martin DD, Binder G. Increase of jump performance during GH treatment in short children born SGA. Front Endocrinol. 2023;14:1122287. 10.3389/fendo.2023.1122287 PMC10153665 37143735

[48] Carrel AL, Myers SE, Whitman BY, Eickhoff J, Allen DB. Long-term growth hormone therapy changes the natural history of body composition and motor function in children with Prader-Willi syndrome. J Clin Endocrinol Metab. 2010;95(3):1131‐1136. 20061431 10.1210/jc.2009-1389 PMC2841537

[49] Reus L, van Vlimmeren LA, Staal JB, Otten BJ, Nijhuis-van der Sanden MWG. The effect of growth hormone treatment or physical training on motor performance in Prader-Willi syndrome: a systematic review. Neurosci Biobehav Rev. 2012;36(8):1817‐1838. 22652271 10.1016/j.neubiorev.2012.05.005

[50] Roemmich JN, Huerta MG, Sundaresan SM, Rogol AD. Alterations in body composition and fat distribution in growth hormone-deficient prepubertal children during growth hormone therapy. Metabolism. 2001;50(5):537‐547. 11319714 10.1053/meta.2001.22510

[51] Hogler W, Briody J, Moore B, Lu PW, Cowell CT. Effect of growth hormone therapy and puberty on bone and body composition in children with idiopathic short stature and growth hormone deficiency. Bone. 2005;37(5):642‐650. 16139578 10.1016/j.bone.2005.06.012

[52] Ferruzzi A, Vrech M, Pietrobelli A, et al The influence of growth hormone on pediatric body composition: a systematic review. Front Endocrinol. 2023;14:1093691. 10.3389/fendo.2023.1093691 PMC9947344 36843617

[53] De Boer H, Blok GJ, Voerman HJ, De Vries PM, van der Veen EA. Body composition in adult growth hormone-deficient men, assessed by anthropometry and bioimpedance analysis. J Clin Endocrinol Metab. 1992;75(3):833‐837. 1517374 10.1210/jcem.75.3.1517374

[54] Badre-Esfahani S, Nellemann B, Danielsen D, Fisker S, Christiansen JS, Jørgensen JO. Assessment of hydration by means of bioelectrical impedance and arm muscle area during growth hormone (GH) replacement therapy: a prospective study of 130 GH-deficient patients. Growth Horm IGF Res. 2007;17(3):227‐233. 17347010 10.1016/j.ghir.2007.01.012

[55] Correia AS, Zymbal V, Baptista F. Musculoskeletal fitness: relative handgrip strength and vertical jump power from 10 to 18 years old. Front Pediatr. 2024;12:1207609. 38333086 10.3389/fped.2024.1207609 PMC10850334

[56] Kim JH, Thompson LV. Differential effects of mild therapeutic exercise during a period of inactivity on power generation in soleus type I single fibers with age. J Appl Physiol. 2012;112(10):1752‐1761. 22422796 10.1152/japplphysiol.01077.2011 PMC3365410

[57] Saeman MR, DeSpain K, Liu MM, et al Effects of exercise on soleus in severe burn and muscle disuse atrophy. J Surg Res. 2015;198(1):19‐26. 26104324 10.1016/j.jss.2015.05.038 PMC4542145

[58] Webb SM, de Andrés-Aguayo I, Rojas-García R, et al Neuromuscular dysfunction in adult growth hormone deficiency. Clin Endocrinol. 2003;59(4):450‐458. 10.1046/j.1365-2265.2003.01866.x 14510907

[59] Chikani V, Cuneo RC, Hickman I, Ho KK. Growth hormone (GH) enhances anaerobic capacity: impact on physical function and quality of life in adults with GH deficiency. Clin Endocrinol. 2016;85(4):660‐668. 10.1111/cen.13147 27346880

[60] Sjögren K, Leung KC, Kaplan W, Gardiner-Garden M, Gibney J, Ho KK. Growth hormone regulation of metabolic gene expression in muscle: a microarray study in hypopituitary men. Am J Physiol Endocrinol Metab. 2007;293(1):E364‐E371. 17456639 10.1152/ajpendo.00054.2007

[61] Hennessey JV, Chromiak JA, DellaVentura S, et al Growth hormone administration and exercise effects on muscle fiber type and diameter in moderately frail older people. J Am Geriatr Soc. 2001;49(7):852‐858. 11527474 10.1046/j.1532-5415.2001.49173.x

[62] Vandoni M, Correale L, Puci MV, et al Six minute walk distance and reference values in healthy Italian children: a cross-sectional study. PLoS One. 2018;13(10):e0205792. Erratum in: PLoS One. 2018 Nov 21;13(11):e0208179. DOI: 10.1371/journal.pone.0208179. 30321226 10.1371/journal.pone.0205792 PMC6188863

[63] Solway S, Brooks D, Lacasse Y, Thomas S. A qualitative systematic overview of the measurement properties of functional walk tests used in the cardiorespiratory domain. Chest. 2001;119(1):256‐270. 11157613 10.1378/chest.119.1.256

[64] Myers J, Prakash M, Froelicher V, Do D, Partington S, Atwood JE. Exercise capacity and mortality among men referred for exercise testing. N Engl J Med. 2002;346(11):793‐801. 11893790 10.1056/NEJMoa011858

[65] Ruiz JR, Castro-Piñero J, Artero EG, et al Predictive validity of health-related fitness in youth: a systematic review. Br J Sports Med. 2009;43(12):909‐923. 19158130 10.1136/bjsm.2008.056499

[66] LeMura LM, von Duvillard SP, Cohen SL, et al Treadmill and cycle ergometry testing in 5- to 6-year-old children. Eur J Appl Physiol. 2001;85(5):472‐478. 11606017 10.1007/s004210100461

[67] Janssen I, Leblanc AG. Systematic review of the health benefits of physical activity and fitness in school-aged children and youth. Int J Behav Nutr Phys Act. 2010;7(1):40. 20459784 10.1186/1479-5868-7-40 PMC2885312

[68] Geisler A, Lass N, Reinsch N, et al Quality of life in children and adolescents with growth hormone deficiency: association with growth hormone treatment. Horm Res Paediatr. 2012;78(2):94‐99. 22907471 10.1159/000341151

[69] Young DR, Hivert MF, Alhassan S, et al Sedentary behavior and cardiovascular morbidity and mortality: a science advisory from the American Heart Association. Circulation. 2016;134(13):e262‐e279. 27528691 10.1161/CIR.0000000000000440

[70] Ekelund U, Steene-Johannessen J, Brown WJ, et al Does physical activity attenuate, or even eliminate, the detrimental association of sitting time with mortality? A harmonised meta-analysis of data from more than 1 million men and women. Lancet. 2016;388(10051):1302‐1310.Erratum in: Lancet. 2016 Sep 24;388(10051):e6. DOI: 10.1016/S0140-6736(16)31677-4. 27475271 10.1016/S0140-6736(16)30370-1

[71] Alkan F, Ersoy B, Kızılay DO, Ozyurt BC, Coskun S. Evaluation of cardiac structure, exercise capacity and electrocardiography parameters in children with partial and complete growth hormone deficiency and their changes with short term growth hormone replacement therapy. Pituitary. 2023;26(1):115‐123. 36463549 10.1007/s11102-022-01295-z

[72] Hagstromer M, Ainsworth BE, Oja P, Sjostrom M. Comparison of a subjective and an objective measure of physical activity in a population sample. J Phys Act Health. 2010;7(4):541‐550. 20683097 10.1123/jpah.7.4.541

[73] Leonardi B, Gentili F, Perrone MA, et al Cardiopulmonary exercise testing in repaired tetralogy of Fallot: multiparametric overview and correlation with cardiac magnetic resonance and physical activity level. J Cardiovasc Dev Dis. 2022;9(1):26. 35050237 10.3390/jcdd9010026 PMC8778451

[74] Quitmann J, Bloemeke J, Silva N, et al Quality of life of short-statured children born small for gestational age or idiopathic growth hormone deficiency within 1 year of growth hormone treatment. Front Pediatr. 2019;7:164. 31111024 10.3389/fped.2019.00164 PMC6501464

[75] Tanaka T, Hasegawa T, Ozono K, et al Effect of growth hormone treatment on quality of life in Japanese children with growth hormone deficiency: an analysis from a prospective observational study. Clin Pediatr Endocrinol. 2014;23(3):83‐92. 25110392 10.1297/cpe.23.83 PMC4125600

[76] Butler G, Turlejski T, Wales G, Bailey L, Wright N. Growth hormone treatment and health-related quality of life in children and adolescents: a national, prospective, one-year controlled study. Clin Endocrinol. 2019;91(2):304‐313. 10.1111/cen.14011 31077606

[77] Shemesh-Iron M, Lazar L, Lebenthal Y, et al Growth hormone therapy and short stature-related distress: a randomized placebo-controlled trial. Clin Endocrinol. 2019;90(5):690‐701. 10.1111/cen.13944 30721549

[78] Stephen MD, Varni JW, Limbers CA, et al Health-related quality of life and cognitive functioning in pediatric short stature: comparison of growth-hormone-naïve, growth-hormone-treated, and healthy samples. Eur J Pediatr. 2011;170(3):351‐358. 20886355 10.1007/s00431-010-1299-z

[79] Maghnie M, Orso M, Polistena B, et al Quality of life in children and adolescents with growth hormone deficiency and their caregivers: an Italian survey. J Endocrinol Invest. 2023;46(12):2513‐2523. 37209402 10.1007/s40618-023-02106-3 PMC10632207

[80] Marra M, Sammarco R, De Lorenzo A, et al Assessment of body composition in health and disease using bioelectrical impedance analysis (BIA) and dual energy X-ray absorptiometry (DXA): a critical overview. Contrast Media Mol Imaging. 2019;2019:3548284. 31275083 10.1155/2019/3548284 PMC6560329

